# The Evaluation of Wound Healing Following Periodontal Flap Closure Using Interrupted Suturing Technique: A Prospective Clinical Study

**DOI:** 10.7759/cureus.115224

**Published:** 2026-08-26

**Authors:** Kratee Sharma, Shalabh Mehrotra

**Affiliations:** 1 Department of Periodontics, Teerthanker Mahaveer Dental College and Research Centre, Teerthanker Mahaveer University, Moradabad, IND

**Keywords:** and periodontal diseases, periodontal flap, periodontal surgical procedures, sutures, wound healing

## Abstract

Introduction

Successful periodontal flap surgery depends on stable wound closure and uncomplicated soft-tissue healing. Appropriate suturing techniques play a crucial role in flap stabilization, primary wound healing, and the reduction of postoperative discomfort, thereby influencing the overall success of periodontal therapy. This study aimed to evaluate wound healing following periodontal flap closure using an interrupted suturing technique by assessing the healing score, plaque index, probing pocket depth, and postoperative pain.

Materials and methods

This prospective clinical study included 30 systemically healthy patients requiring periodontal flap surgery. Following Phase I periodontal therapy, periodontal flap surgery was performed, and the flaps were repositioned and secured using the interrupted suturing technique. The primary outcome was wound healing, assessed using the Early Wound Healing Score (EHS) on postoperative days three, seven, and 14. Secondary outcomes included the plaque index and probing pocket depth, recorded at baseline and at two weeks, and postoperative pain, assessed using a 10-cm Visual Analog Scale (VAS) on postoperative days three, seven, and 14. The Wilcoxon signed-rank test was used for paired comparisons, while the Friedman test followed by a Bonferroni-adjusted post-hoc analysis was used for repeated measures. Statistical significance was set at p < 0.05.

Results

The study included 30 participants with a mean age of 35.03 ± 6.68 years. The plaque index significantly decreased from 1.32 (interquartile range (IQR): 1.16-2.00) at baseline to 0.53 (IQR: 0.32-0.67) at day 14 (p < 0.001). The probing pocket depth significantly decreased from 3.63 mm (IQR: 3.43-3.72) to 2.38 mm (IQR: 1.97-2.45) (p < 0.001). The EHS scores improved progressively from 6.00 (IQR: 6.00-7.00) on day three to 9.00 (IQR: 8.00-9.00) on day 14 (χ² = 53.51, p = 0.001), while the median VAS scores decreased significantly from 7.00 (IQR: 6.25-7.00) to 3.00 (IQR: 2.00-4.00) during the same period (χ² = 60.00, p = 0.001). Bonferroni-adjusted post-hoc analysis demonstrated significant differences between all postoperative time points for both healing and pain scores.

Conclusions

Periodontal flap closure using the interrupted suturing technique was associated with favorable wound healing, reductions in plaque index and probing pocket depth, decreased postoperative pain, and progressive improvement in soft-tissue healing. Interrupted suturing may provide satisfactory flap adaptation and can be considered a practical method for flap closure following periodontal flap surgery; however, comparative studies are required to determine its relative effectiveness compared with other closure techniques.

## Introduction

Periodontal flap surgery is a well-established therapeutic procedure for managing chronic periodontitis, providing direct access for thorough root debridement and elimination of periodontal pockets. Successful outcomes after flap surgery depend not only on meticulous surgical technique but also on effective flap approximation and stabilization during healing [1]. Precise wound closure minimizes dead space, controls bleeding, protects underlying tissues from microbial contamination, and facilitates primary intention healing, thereby enhancing postoperative comfort and tissue regeneration [2].

Suturing remains the most commonly employed method for periodontal flap closure because it provides adequate tissue adaptation while maintaining the flap in its desired position throughout the early phases of healing [3]. Among the various suturing techniques available, the interrupted suturing technique is widely practiced owing to its simplicity, ease of placement, excellent flap stabilization, and ability to maintain wound closure even if one suture fails [4]. Appropriate suturing techniques influence several clinical outcomes, including plaque accumulation, postoperative inflammation, wound healing, probing pocket reduction, and patient-reported pain [5]. Therefore, evaluating the effectiveness of suturing techniques is essential to optimize clinical outcomes and improve patient satisfaction.

Postoperative healing following periodontal surgery is dynamic and can be assessed using both objective and subjective parameters. Clinical indices such as the plaque index and probing pocket depth provide information regarding periodontal health and treatment effectiveness, whereas healing indices evaluate soft tissue repair [6]. Additionally, patient-reported pain measured using the Visual Analog Scale (VAS) offers valuable insight into postoperative discomfort and recovery [7]. Monitoring these parameters at different postoperative intervals enables clinicians to comprehensively evaluate the biological and clinical success of periodontal flap surgeries.

The primary objective of this prospective clinical study was to describe the temporal course of wound healing following periodontal flap surgery closed with interrupted sutures by assessing the Early Wound Healing Score (EHS) during the postoperative period. The secondary objectives were to describe changes in plaque index and probing pocket depth from baseline to two weeks after surgery and to assess the temporal pattern of postoperative pain intensity using the VAS on postoperative days three, seven, and 14.

## Materials and methods

Study design and setting

This prospective clinical study was conducted at the Department of Periodontics, Kothiwal Dental College and Research Centre, Moradabad, Uttar Pradesh, India, from July 2025 to December 2025. To minimize operator-related variability, all surgical procedures and postoperative clinical assessments were performed under standardized clinical conditions by the same experienced periodontist. Eligible patients requiring periodontal flap surgery were consecutively recruited from the outpatient department throughout the study period until the predetermined sample size was reached.

Ethical considerations

Prior to commencement of the study, the study protocol was reviewed and approved by the Institutional Ethics Review Board of the college (KDCRC/IERB/04/2025/13, dated June 19, 2025). The study was conducted in accordance with the ethical principles outlined in the 2013 revision of the Declaration of Helsinki for research involving human participants. Before enrollment, each eligible participant received a detailed explanation of the study objectives, surgical procedure, expected benefits, potential risks, and follow-up schedule. Written informed consent was obtained from all participants before the initiation of any study-related procedures. Patient confidentiality was maintained throughout the study, and participants were free to withdraw at any time without affecting their treatment.

Patient selection

Patients diagnosed with Stage III or Stage IV periodontitis, according to the 2017 World Workshop Classification of Periodontal and Peri-Implant Diseases and Conditions, and requiring periodontal flap surgery were screened for eligibility [8]. The diagnosis was established based on the presence of probing pocket depths ≥ 5 mm, clinical attachment loss, radiographic evidence of alveolar bone loss, and persistent periodontal pockets requiring surgical intervention following completion of Phase I periodontal therapy. Only systemically healthy individuals aged 25-50 years who demonstrated satisfactory plaque control after nonsurgical periodontal therapy and were willing to comply with the postoperative follow-up schedule were included in the study. Patients with uncontrolled systemic diseases known to influence periodontal wound healing, those who smoked or used tobacco, pregnant or lactating women, individuals receiving antibiotics, corticosteroids, immunosuppressive agents, or anticoagulant therapy within the preceding three months, patients who had undergone periodontal surgery at the intended surgical site within the previous six months, and those with inadequate oral hygiene compliance or an inability to attend scheduled follow-up visits were excluded from the study.

Sample size estimation

The sample size was estimated using G*Power software version 3.1.9 (Heinrich Heine University Düsseldorf, Düsseldorf, Germany). Based on the magnitude of postoperative changes in early wound healing reported in the periodontal surgical literature, a moderate-to-large anticipated effect size of 0.60 was selected for the primary outcome, EHS [9]. With a statistical power of 80% and a two-sided significance level of 0.05, the minimum required sample size was estimated to be 27 participants. Considering an anticipated attrition rate of approximately 10%, the final sample size was increased to 30 participants. The primary outcome was assessed repeatedly on postoperative days three, seven, and 14, and temporal changes in EHS were evaluated using the Friedman test with Bonferroni-adjusted post-hoc comparisons.

Clinical procedure

All participants initially underwent Phase I periodontal therapy, which included oral hygiene instructions, supragingival and subgingival scaling, and root planing. Surgical intervention was scheduled only after satisfactory plaque control had been achieved. Periodontal flap surgery was performed at sites with persistent periodontal pockets requiring surgical access following completion of Phase I therapy. The surgical sites were clinically evaluated before intervention to confirm the need for flap surgery. The procedure was performed under local anesthesia using 2% lignocaine hydrochloride with 1:80,000 epinephrine (LOX 2% Adrenaline; Neon Laboratories Ltd., Mumbai, Maharashtra, India). Intracrevicular incisions were made around the involved teeth using a No. 15 Bard-Parker surgical blade (Swann-Morton Ltd., Sheffield, United Kingdom), and full-thickness mucoperiosteal flaps were carefully reflected using a periosteal elevator to provide adequate access for debridement. Granulation tissue was meticulously removed, and scaling and root planing were completed using Gracey curettes until smooth root surfaces were obtained. Before flap repositioning, the surgical field was thoroughly irrigated with sterile normal saline to eliminate residual debris and blood clots.

**Figure 1:**
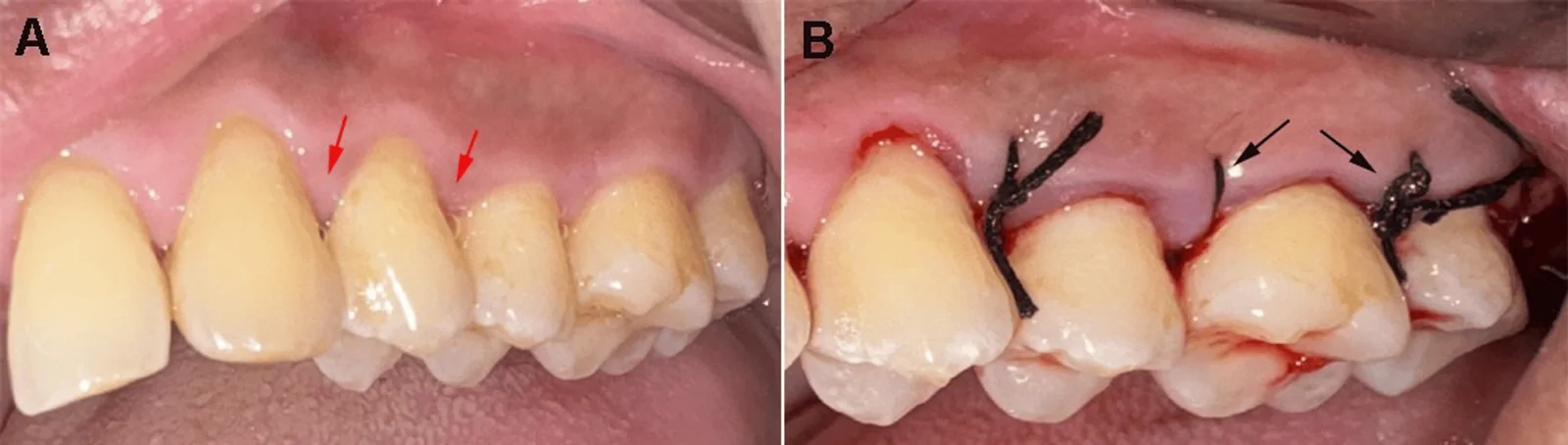
Pre- and immediate postoperative clinical photographs (A) Preoperative clinical photograph of gingival contour (red arrow). (B) Immediate postoperative clinical photograph showing flap closure using interrupted sutures (black arrows)

Following debridement, the periodontal flaps were repositioned to their original anatomical position and secured using interrupted 3-0 black braided silk sutures (MERSILK®, Ethicon, Inc., Raritan, NJ). Individual interrupted sutures were placed at approximately uniform intervals along the surgical wound, with the number of sutures determined by the extent and length of the surgical site to ensure adequate flap adaptation. Care was taken to achieve passive approximation of the wound margins without excessive tissue tension. The same surgical approach, including flap reflection, debridement, irrigation, flap repositioning, and interrupted suturing, was followed for all participants. Hemostasis was confirmed before completion of the surgical procedure (Figure 1).

Postoperative care and outcome assessment

**Figure 2:**
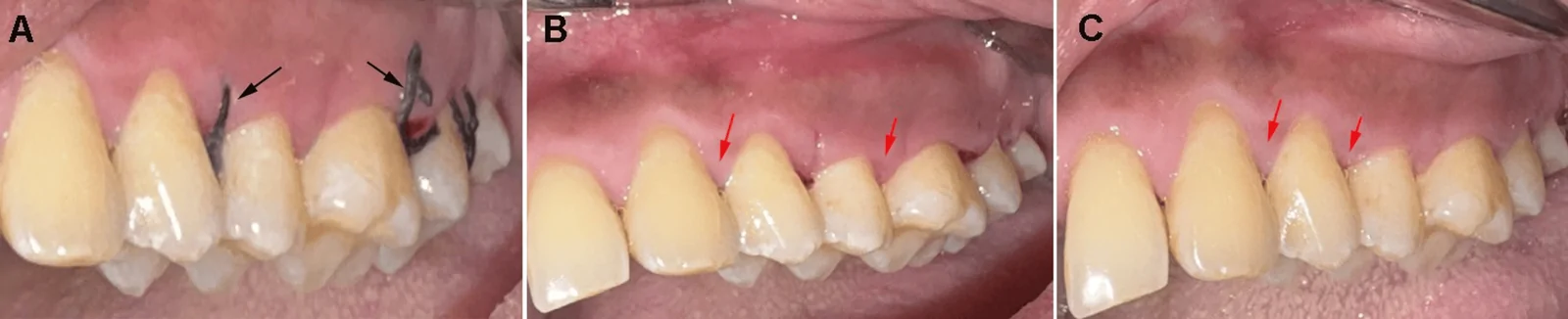
Postoperative follow-up images Postoperative follow-up images demonstrating healing at (A) postoperative day three, (B) postoperative day seven, and (C) postoperative day 14. Black arrows: suture threads; red arrows: healed area

Following surgery, all participants received standardized postoperative instructions regarding oral hygiene maintenance, dietary precautions, and surgical site care. For postoperative pain management, ibuprofen 400 mg was prescribed orally every eight hours for three days. Participants were instructed to take the medication as prescribed during the initial postoperative period and to record any additional analgesic use. No other routine analgesic medication was prescribed. Any additional or rescue analgesic use during the follow-up period was documented and considered during interpretation of postoperative VAS scores. The same analgesic protocol was followed for all participants to minimize variation in analgesic exposure. Patients were recalled on postoperative days three, seven, and 14 for the evaluation of early wound healing and postoperative pain, while plaque index and probing pocket depth were reassessed two weeks after surgery. The sutures were removed after satisfactory initial wound healing was achieved (Figure 2).

Clinical assessments were performed by a calibrated examiner using a University of North Carolina-15 (UNC-15) periodontal probe (Hu-Friedy Mfg. Co., LLC, Chicago, IL). Before commencement of the study, the examiner underwent calibration for assessment of the EHS using representative postoperative clinical cases. The calibration was performed by repeated assessment of the same cases at a predetermined interval to ensure consistency in scoring. Intra-rater reliability for EHS assessment was evaluated using the intraclass correlation coefficient (ICC), which demonstrated good-to-excellent agreement. The examiner was blinded to the previous scores during the repeat assessment. The primary outcome measure of the study was early wound healing assessed using the EHS described by Marini et al. [10], which assesses three clinical domains, yielding a total score ranging from 0 to 10, with higher scores indicating better early wound healing. Secondary outcome measures included plaque index [11] and probing pocket depth recorded at baseline and two weeks after surgery, together with postoperative pain intensity assessed using a 10-cm VAS on postoperative days three, seven, and 14 [12]. VAS and EHS are distributed under the terms of the Creative Commons License (CC BY 4.0, CC BY-NC 4.0). 

Statistical analysis

The collected data were analyzed using IBM SPSS Statistics version 26 (IBM Corp., Armonk, NY). Continuous variables were summarized using descriptive statistics. Data distribution was assessed using the Shapiro-Wilk test, which demonstrated that the plaque index, probing pocket depth, EHS, and VAS scores were not normally distributed. Consequently, non-parametric statistical tests were used for analysis. Comparisons between baseline and two-week values for plaque index and probing pocket depth were performed using the Wilcoxon signed-rank test, whereas changes in EHS and VAS scores on postoperative days three, seven, and 14 were evaluated using the Friedman test, followed by pairwise Wilcoxon signed-rank tests with Bonferroni adjustment for multiple comparisons. The reported p-values for the pairwise comparisons were Bonferroni-adjusted. Statistical significance was established at a two-tailed p < 0.05.

## Results

Thirty participants were included in the study. Of these, 22 (73.3%) were diagnosed with Stage III periodontitis, while eight (26.7%) had Stage IV periodontitis. The mean age of the study population was 35.03 ± 6.68 years (range: 28-50 years). The sample comprised 17 (56.7%) females and 13 (43.3%) males, indicating a relatively balanced distribution by sex (Table 1).

**Table 1:** Demographic characteristics of the study participants SD: standard deviation

Characteristic	Value
Total number of subjects	30
Age, years, mean ± SD	35.03 ± 6.68
Age range, years	28–50
Male, n (%)	13 (43.3%)
Female, n (%)	17 (56.7%)

A significant improvement in periodontal clinical parameters was observed following periodontal flap surgery. Comparison of baseline and two-week measurements demonstrated a marked reduction in both plaque index and probing pocket depth. The median plaque index decreased from 1.32 (interquartile range (IQR): 1.16-2.00) at baseline to 0.53 (IQR: 0.32-0.67) at day 14. Similarly, the median probing pocket depth reduced from 3.63 mm (IQR: 3.43-3.72) to 2.38 mm (IQR: 1.97-2.45). Wilcoxon signed-rank analysis confirmed that both reductions were statistically significant (p < 0.001), with large effect sizes, indicating substantial clinical improvement following treatment (Table 2).

**Table 2:** Comparison of plaque index and probing pocket depth between baseline and postoperative day 14 ^*^P < 0.05 was considered statistically significant Comparisons were performed using the Wilcoxon signed-rank test; effect size is reported as r IQR: interquartile range

Parameter	Baseline median (IQR)	Day 14 median (IQR)	z-value	P-value	Effect size
Plaque index (PI)	1.32 (1.16-2.00)	0.53 (0.32-0.67)	4.79	< 0.001^*^	0.87
Probing pocket depth (mm)	3.63 (3.43-3.72)	2.38 (1.97-2.45)	4.79	< 0.001^*^	1.00

Evaluation of the postoperative wound healing revealed progressive improvement throughout the follow-up period. The median EHS increased from 6.00 (IQR: 6.00-7.00) on postoperative day three to 8.00 (IQR: 8.00-8.00) on day seven, and further to 9.00 (IQR: 8.00-9.00) on day 14. Friedman test analysis demonstrated that these improvements were statistically significant (χ² = 53.51, p = 0.001), with a large effect size (0.89). Conversely, postoperative pain showed a continuous decline during the healing period, with median VAS scores decreasing from 7.00 (IQR: 6.25-7.00) on day three to 5.00 (IQR: 4.00-5.00) on day seven and 3.00 (IQR: 2.00-4.00) on day 14. The reduction in pain was also statistically significant (χ² = 60.00, p = 0.001), with an effect size of 1.00 (Table 3).

**Table 3:** Comparison of EHS and VAS scores at different postoperative time points ^*^P < 0.05 was considered statistically significant Comparisons were performed using the Friedman test; effect size was reported as Kendall's W EHS: Early Wound Healing Score; VAS: Visual Analog Scale; IQR: interquartile range

Parameter	Time point	Median (IQR)	Test value (Friedman)	P-value	Effect size
Healing score (EHS)	Day 3	6.00 (6.00–7.00)	53.51	0.001^*^	0.89
	Day 7	8.00 (8.00–8.00)			
	Day 14	9.00 (8.00–9.00)			
VAS score	Day 3	7.00 (6.25–7.00)	60.00	0.001^*^	1.00
	Day 7	5.00 (4.00–5.00)			
	Day 14	3.00 (2.00–4.00)			

Post-hoc pairwise comparisons using Bonferroni adjustment demonstrated statistically significant differences between all postoperative time points for both the EHS and pain scores. EHS improved significantly between postoperative days three and seven, seven and 14, and three and 14 (all p < 0.05). Likewise, VAS scores showed significant reductions between each consecutive follow-up interval, as well as between days three and 14 (all p < 0.001), indicating continuous wound healing accompanied by progressive reduction in postoperative pain during the first two weeks following periodontal flap surgery (Table 4).

**Table 4:** Post-hoc pairwise comparison of EHS and VAS scores between postoperative time points ^*^P < 0.05 was considered statistically significant Pairwise comparisons were performed using the Wilcoxon signed-rank test following the Friedman test EHS: Early Wound Healing Score; VAS: Visual Analog Scale

Comparison	Healing score (EHS)			VAS		
	Mean difference	Z-value	P-value	Mean difference	Z-value	P-value
Day 3 vs. day 7	-1.30	-4.01	< 0.001^*^	2.03	3.87	< 0.001^*^
Day 7 vs. day 14	-1.00	-6.84	< 0.001^*^	2.07	7.75	< 0.001^*^
Day 3 vs. day 14	-2.30	-2.84	< 0.014^*^	4.10	3.87	< 0.001^*^

**Figure 3:**
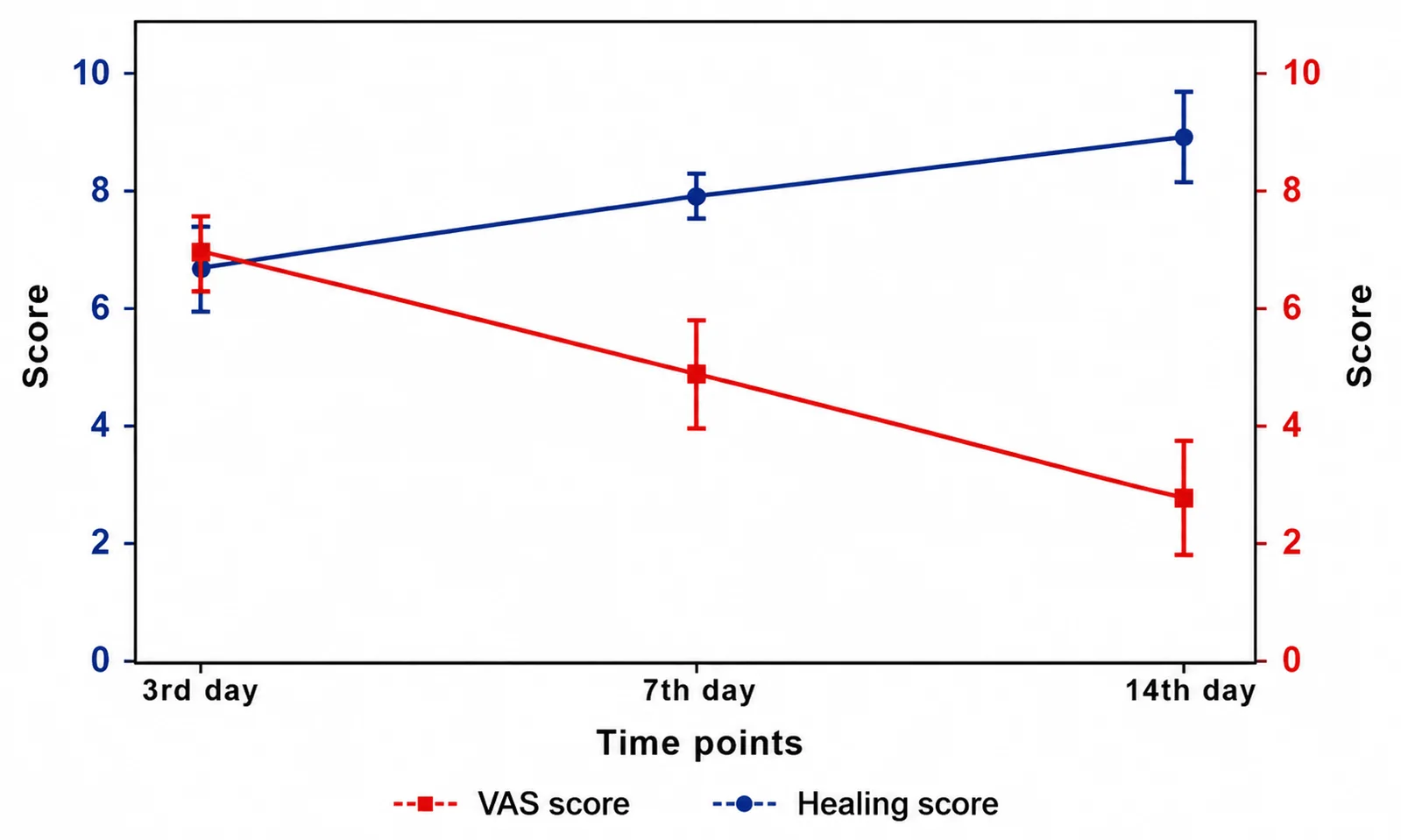
Trend of mean healing score (blue line) and mean VAS score (red line) across day three, day seven, and day 14 Error bars: standard deviation. Healing score: EHS VAS: Visual Analog Scale; EHS: Early Wound Healing Score

Figure 3 illustrates postoperative trends in wound healing and pain intensity. The mean EHS increased steadily from day three to 14, whereas the mean VAS score demonstrated a corresponding progressive decline over the same period, reflecting favorable postoperative healing and improved patient comfort.

## Discussion

The present prospective clinical study evaluated wound healing following periodontal flap surgery with interrupted suture closure by assessing changes in plaque index, probing pocket depth, wound healing, and postoperative pain. The findings demonstrated significant improvements in all evaluated clinical parameters during the postoperative period. The progressive increase in EHS, reduction in postoperative pain, and improvement in periodontal parameters indicate favorable postoperative healing and recovery following periodontal flap surgery with interrupted sutures. However, because the study did not include a comparator group, these improvements cannot be attributed specifically to the interrupted suturing technique and may reflect the combined effects of the surgical procedure, periodontal treatment, postoperative care, and the natural course of wound healing.

The study demonstrated a significant reduction in the plaque index from baseline to two weeks after surgery. This finding is clinically important because plaque control plays a pivotal role in preventing bacterial colonization of surgical wounds and facilitating uneventful periodontal healing [13]. Following periodontal therapy, patients generally exhibit improved oral hygiene practices due to repeated reinforcement of plaque control measures and increased motivation during follow-up visits [14]. Therefore, the decrease in plaque accumulation observed in the present study may be attributed to meticulous oral hygiene instructions, professional debridement before surgery, and enhanced patient compliance during the healing period.

These findings are consistent with those reported by Silness and Löe [15], who demonstrated that effective plaque control is fundamental for maintaining gingival health and promoting periodontal healing following surgical intervention. Similarly, Rosling et al. [16] reported that patients who maintained optimal plaque control after periodontal surgery exhibited superior healing and greater long-term periodontal stability than those with inadequate plaque control. The present findings further support the concept that successful surgical outcomes depend not only on the surgical procedure itself but also on effective postoperative plaque control.

A significant reduction in probing pocket depth was also observed two weeks after surgery. Reduction in periodontal pocket depth represents one of the principal therapeutic objectives of periodontal flap surgery because it reflects the successful elimination of inflamed pocket epithelium, improved root surface debridement, and favorable adaptation of gingival tissues during healing [17]. Following open flap debridement, removal of granulation tissue and bacterial deposits permits the resolution of inflammation, resulting in gingival shrinkage and re-establishment of a healthier periodontal attachment [18]. Consequently, the reduction in probing depth observed in the present study indicates a favorable periodontal tissue response following flap surgery.

These observations are in agreement with the classic investigations of Kaldahl et al. [19], who reported significant reductions in probing depth following flap surgery combined with meticulous root planing. Similar improvements have also been documented by Lindhe and Nyman [20], who demonstrated that periodontal flap procedures effectively reduced periodontal pocket depth and improved clinical attachment when combined with adequate postoperative maintenance. Therefore, the present study further supports the effectiveness of conventional periodontal flap surgery in improving periodontal health.

One of the most important findings of the present investigation was the progressive improvement in EHS during the first two postoperative weeks. The healing scores increased significantly from day three to day 14, indicating continuous progression of surgical wound healing. Early wound healing following periodontal surgery depends on stable flap approximation, adequate vascular perfusion, minimal tissue trauma, and protection of the wound from bacterial contamination. Interrupted sutures provide independent stabilization of the individual segments of the flap, thereby minimizing flap displacement and facilitating healing by primary intention [9]. The present findings are consistent with those of Burkhardt and Lang [21], who reported that proper flap adaptation and stabilization through appropriate suturing are fundamental for fibrin clot stability and optimal periodontal wound healing.

The satisfactory wound healing achieved in the present study with 3-0 black braided silk sutures is in agreement with Korukonda et al. [22], who demonstrated that silk sutures provide acceptable soft-tissue healing, although the choice of suture material should be individualized according to the surgical procedure and tissue characteristics. The satisfactory healing achieved with the interrupted suturing technique may be attributed to accurate flap adaptation and appropriate tension control. Moy et al. highlighted that a meticulous suturing technique is fundamental for promoting uneventful wound healing and preventing tissue trauma [23].

Postoperative pain is an important patient-centered outcome because it directly influences treatment acceptance and overall satisfaction. In the present study, VAS scores decreased significantly from postoperative day three to day 14, indicating progressive improvement in patient comfort throughout the healing period. Pain following periodontal surgery is generally associated with tissue trauma, release of inflammatory mediators, edema, and mechanical irritation at the surgical site. As inflammation gradually resolves and epithelialization progresses, nociceptive stimulation diminishes, resulting in lower pain scores during subsequent follow-up visits [24].

The observed reduction in pain agrees with the findings of Yuan et al. [9], who reported that postoperative discomfort after periodontal surgery decreases substantially within the first two weeks when appropriate surgical techniques and postoperative care are employed. Similarly, Cortellini and Tonetti [25] demonstrated that atraumatic surgical procedures combined with stable wound closure significantly improved patient comfort and accelerated early wound healing. Therefore, the favorable pain reduction observed in the present study may be attributed to careful tissue handling, secure flap stabilization using interrupted sutures, and adherence to standardized postoperative instructions.

The progressive reduction in postoperative pain observed during the first two weeks should be interpreted cautiously. In addition to the effects of surgical wound closure, pain scores may have been influenced by standardized postoperative analgesic use and the normal biological course of wound healing, including resolution of inflammation, edema, and tissue repair. Therefore, the observed decrease in VAS scores cannot be attributed specifically to the interrupted suturing technique. Rather, it likely reflects the combined effects of postoperative care, analgesic management, natural resolution of the surgical inflammatory response, and progressive wound healing following periodontal flap surgery.

The significant differences observed between all postoperative time points in the Bonferroni-adjusted pairwise analysis further indicated that wound healing was a continuous biological process rather than an abrupt event. Significant improvement from day three to day seven reflects the transition from the inflammatory phase to the proliferative phase of wound healing, which is characterized by fibroblast proliferation, angiogenesis, and collagen deposition. The additional improvement observed between days seven and 14 corresponds to progressive epithelial maturation and connective tissue remodeling [26]. These findings are consistent with the established biological sequence of periodontal wound healing and demonstrate that interrupted sutures maintained adequate wound stability during the critical early healing period.

Overall, the findings of the present study demonstrate that periodontal flap closure using the interrupted suturing technique provides favorable postoperative healing, with significant improvements in periodontal clinical parameters and patient comfort. Although several contemporary suturing techniques and tissue adhesives have been introduced, interrupted sutures remain one of the most reliable and widely practiced methods because of their simplicity, versatility, and ability to provide independent flap stabilization in routine periodontal surgical procedures [3,4].

This study had several important clinical implications. The findings support the continued use of the interrupted suturing technique as a simple, economical, and effective method for achieving stable flap approximation and favorable periodontal wound healing following flap surgery. The significant reduction in plaque accumulation, probing pocket depth, and postoperative pain, together with progressive improvement in EHS, indicate that this technique provides satisfactory clinical outcomes while potentially enhancing patient comfort. However, this study has some limitations. The study included a relatively small sample size from a single institution, which may limit the generalizability of the findings. The absence of a comparison group using an alternative suturing technique or tissue adhesive precludes direct comparison of the effectiveness of different closure methods.

Furthermore, the follow-up period was limited to two weeks for periodontal parameters and wound healing assessment, preventing the evaluation of long-term periodontal stability. Another limitation is that all surgical procedures were performed by a single experienced periodontist. Although this approach minimized inter-operator variability and ensured procedural consistency, the findings may partly reflect the operator's individual surgical and suturing expertise, which may limit the generalizability of the results to other clinicians and clinical settings. In addition, formal intra-rater reliability testing for the EHS was not performed, and this should be considered when interpreting this clinically assessed outcome. Future multicenter randomized clinical trials with larger sample sizes, extended follow-up periods, and direct comparisons between different flap closure techniques are recommended to further validate these findings.

## Conclusions

Within the limitations of this prospective clinical study, periodontal flap closure using the interrupted suturing technique was associated with favorable postoperative outcomes, including progressive improvement in wound healing, reductions in plaque index and probing pocket depth, and decreased postoperative pain during the healing period. These findings suggest that interrupted suturing can provide satisfactory flap adaptation following periodontal flap surgery and may be a practical option for routine surgical wound closure. However, in the absence of a comparison group, the observed improvements cannot be attributed specifically to the interrupted suturing technique.
